# Book Notices

**Published:** 1868-02-15

**Authors:** 


### *BOOK NOTICES.*

---

ANNUAL ABSTRACT OF THERAPEUTICS, MATERIA MEDICA, PHARMACY, AND TOXICOLOGY, FOR 1867; followed by an original memoir on Gout, Gravel, and Urinary Calculi. By A. BOUCHARDAT, Prof. of Hygiene to the Faculty of Medicine, Paris; Member of the Imperial Academy of Medicine. Translated and edited by M. J. DE ROSSET, M.D., Adjunct Prof. of Chemistry, University of Maryland; Member of the Maryland Academy of Sciences. Philadelphia: Lindsay & Blakiston. 1868. Pp. 314.

A very useful little work, giving a *resume* of the important subjects noted in the title, "as they appear in Paris," and in small compass affording "aid and comfort" to the hard-working practitioner whose time and means will not permit investigation of the original sources. A note, with enclosed slip from the publishers, calls attention to an unfortunate error on page 139, which should be corrected at once by every reader of the book. In the formula for the *Liquor Donavan-Ferari*, change it to read as follows: "℞ Iodide of arsenic, 3 grains; distilled water, 30 drachms; mix, and dissolve by heat in a glass mattress; add, biniodide of mercury, 6 grains; iodide of potassium, 45 or 60 grains; filter and preserve in a brown bottle, well corked. The solution thus obtained is limpid, and of a light straw tint; one drachm containing about $\frac{9}{100}$ of a grain of iodide of arsenic, and about double that quantity of biniodide of mercury."

THE HALF-YEARLY ABSTRACT OF THE MEDICAL SCIENCES. Being a digest of British and Continental Medicine, and of the Progress of Medicine and the Collateral Sciences. Vol. XLVI. July–December, 1667. Philadelphia: Henry C. Lea. 1868.

BRAITHWAITE'S RETROSPECT. Part LVI. January, 1868. W. A. TOWNSHEND & ADAMS, 434 Broome street, New York.

HALF YEARLY COMPENDIUM OF MEDICAL SCIENCE. Part I. January, 1868. Philadelphia: Published by the proprietors, 115 South Seventh street. 1868. Edited by Drs. Butler and Brinton.

